# Duration of intensive care unit stay as a guide for the timely treatment of Carbapenem-resistant *Enterobacterales* bloodstream infection: a case-control study

**DOI:** 10.1017/ash.2025.10198

**Published:** 2025-10-20

**Authors:** Eda Karadoğan, Hanife Uzar, Aslı Özden, Büşra Dalgıç, Mervenur Demir, Ahmet Sertçelik, Gülçin Telli Dizman, Banu Çakır, Gülşen Hazırolan, Omrum Uzun, Gökhan Metan

**Affiliations:** 1 Hacettepe University Faculty of Medicine Department of Public Health Division of Epidemiology, Ankara, Türkiye; 2 Hacettepe University Faculty of Medicine, Ankara, Türkiye; 3 Hacettepe University Faculty of Medicine Department of Clinical Microbiology, Ankara, Türkiye; 4 Hacettepe University Faculty of Medicine Department of Infectious Diseases and Clinical Microbiology, Ankara, Türkiye

## Abstract

Timely selection of appropriate empirical treatment for carbapenem-resistant *Enterobacterales* (CRE) bacteremia remains challenging, especially when prior antibiotic exposure data is unavailable. We found that intensive care unit stay duration predicts CRE bacteremia—with ≥8 days showing 81% sensitivity and 96% PPV—providing a practical clue for empirical therapy decisions.

Carbapenem-resistant *Enterobacterales* (CRE) represents a critical global health threat, characterized by its rapid dissemination within healthcare systems and association with high mortality rate.^1^ Our national surveillance data reveal alarmingly high carbapenem resistance in healthcare-associated infections, with rates of 18.35% in Escherichia coli and 70.9% in *Klebsiella pneumoniae*.^2^

Our hospital setting has a high prevalence of CRE.^3^ Among 4,105 patients screened for carbapenem-resistant *Klebsiella pneumoniae* (CR-Kp) at our hospital, 279 (6.8%) were colonized.^4^ Rectal swab cultures detected colonization in 25% of patients in oncology and internal medicine wards, including 10% of patients who were colonized within 48 hours of ICU admission (Hacettepe University Hospital Infection Control Committee records, unpublished data).

Antimicrobial exposure and CRE colonization remain the principal risk factors for CRE infections.^5^ However, critical gaps in clinical documentation during interhospital transfers may significantly hinder effective risk stratification. Discharge summaries frequently lack essential details of antibiotic histories—including specific agent classes, treatment durations, and sequencing patterns—while consecutive or overlapping antibiotic regimens further obscure individual drug contributions to CRE. Additionally, screening for CRE colonization is not universally performed.^6^ Prolonged ICU admission increases the likelihood of exposure to broad-spectrum antibiotics and the risk of CRE cross-contamination, particularly in endemic settings.

In this study, we focused on intensive care unit (ICU) admission as a readily identifiable risk factor for CRE infections. This was a case-control study. Cases were defined as patients with bloodstream infections caused by CRE, while controls were patients with bloodstream infections caused by extended-spectrum beta-lactamase producing Enterobacterales (ESBL-E). This study was conducted at Hacettepe University Hospitals, a 1,100-bed tertiary care facility, and evaluated data from adult patients (≥18 yr) between 2017 and 2020. Demographic and clinical information was collected retrospectively from the hospital information system [Approved by Hacettepe University Non-Interventional Clinical Research Ethics Board (2021/13–18), (2023/23–579)]. Univariate and multivariate analyses were performed to identify risk factors associated with CRE. A ROC curve was plotted to identify the day of admission at which the risk of CRE increased for patients in the ICU. The cutoff point was determined using the Youden index. A lower cutoff point was also considered in order to enhance sensitivity.

After excluding 90 patients with polymicrobial bloodstream infections, the study included 134 patients with CRE bacteremia (*K. pneumoniae* 85.1%, *E. coli* 6.7%, *Enterobacter cloacae* 5.2%, *Klebsiella oxytoca* 2.2%, and *Citrobacter freundii* 0.7%) and 115 patients with ESBL-E bacteremia (*E. coli* 77.4%, and *K. pneumoniae* 22.6%). Admission rate to ICU and duration of hospitalization in ICU was higher in patients with CRE bacteremia (Table 1). In multivariate analysis, after adjusting for history of ICU admission prior to bacteremia, patients with CRE bacteremia had significantly higher odds ratio of being in the ICU at the time of bacteremia occurrence compared to those with ESBL- E [adjusted OR = 6.4, 95% CI = 2.9 – 14.0, *P* < .001].

**Table 1.** Demographic and clinical characteristics of the patients with carbapenem-resistant Enterobacterales bacteremia, compared to those with extended-spectrum beta-lactamase (ESBL) producing Enterobacterales

	Patients with ESBL-producing Enterobacterales	Patients with Carbapenem-Resistant Enterobacterales	OR (95% CI)	*p* value
Age (years), Median (IQR)	61 (46 – 70)	64 (50 – 74)		.319
Female, *n* (%)^a^	59 (51.3)	63 (47.0)	.8 (.5 – 1.4)	.500
Being in the intensive care unit when bacteremia occurred, n (%)^a^	11 (9.6)	62 (46.3)	8.1 (4.0 – 16.5)	<**.001**
History of ICU stay prior to bacteremia, n (%)^a^	33 (28.7)	79 (59.9)	3.6 (2.1 – 6.1)	**<.001**
Duration of ICU stay prior to bacteremia (days)^b^, Median (IQR)	8 (4 – 15)	14 (5 – 31)		**.044**
Comorbidities, *n* (%)^a^				
Hematological malignancy	25 (21.7)	34 (25.4)	1.2 (.7 – 2.2)	.501
Solid tumor	39 (33.9)	44 (32.8)	1.0 (.6 – 1.6)	.857
Congestive heart failure	12 (10.4)	13 (9.7)	.9 (.4 – 2.1)	.848
Diabetes mellitus	28 (24.3)	33 (24.6)	1.0 (.6 – 1.8)	.959
Hypertension	31 (27.0)	59 (44.0)	2.1 (1.2 – 3.6)	**.005**
Chronic obstructive pulmonary disease	2 (1.7)	14 (10.4)	6.6 (1.5 – 29.7)	**.005**
Connective tissue disease	4 (3.5)	4 (3.0)	.9 (.2 – 3.5)	.826
Chronic liver disease	10 (8.7)	8 (6.0)	.7 (.3 – 1.8)	.408
Chronic kidney disease	17 (14.8)	15 (11.2)	.7 (.3 – 1.5)	.399
Hematopoeteic stem cell transplantation, *n* (%)^a^	6 (5.2)	13 (9.7)	2.0 (.7 – 5.3)	.184
Graft versus host disease, *n* (%)^a^	1 (.9)	2 (1.5)	1.7 (.2 – 19.3)	1.000
History of Chemotherapy, *n* (%)^a^	34 (29.6)	44 (32.8)	1.2 (.7 – 2.0)	.579
Neutropenia (<500 mm^3^), *n* (%)^a^	24 (20.9)	31 (23.1)	1.1 (.6 – 2.1)	.668
Surgery in the previous 3 months,* n* (%)^a^	28 (24.3)	31 (23.1)	.9 (.5 – 1.7)	.822
Hospital stay of 72 hours or more	107 (93.0)	124 (92.5)	.9 (.4 – 2.4)	.878
Immunosuppressive therapy in the last 1 month/neutropenic/postallogeneic hematopoietic stem cells transplant (allogeneic *HSCT*) *<* 1year/untreated hematological malignancy, *n* (%)^a^	52 (45.2)	50 (37.3)	.7 (.4 – 1.2)	.206
Hemodialysis, *n* (%)^a^	14 (12.2)	12 (9.0)	.7 (.3 – 1.6)	.408
The suspected source of infection,* n* (%)^a^				
Pneumonia	12 (10.4)	21 (15.7)		**<.001**
Catheter-related bacteremia	23 (20.0)	1 (.7)		
Urinary tract infection	22 (19.1)	20 (14.9)		
Intraabdominal infection	31 (27.0)	13 (9.7)		
Febrile neutropenia	14 (12.2)	31 (23.1)		
Unknown source of bacteremia	10 (8.7)	42 (31.3)		
Others^c^	3 (2.6)	6 (4.5)		

ESBL, Extended spectrum beta-lactamase; OR, Odds ratio; CI, Confidence interval; IQR, Interquartile range; ICU, Intensive Care Unit; HSCT, Hematopoietic stem cell transplantation; MDR, Multidrug-resistant.

a Column percent, ^b^It was analyzed in 112 patients with a history of ICU admission,^c^ Wound infection (*n* = 7), central nervous system infection (*n* = 2).

The ROC curve analysis demonstrated moderate predictive accuracy for ICU stay duration in identifying CRE bacteremia (AUC = 0.704, 95% CI = 0.503–0.904; *P* = .043). Using the Youden index, we identified an optimal cutoff of ≥13 days of ICU stay to predict CRE bacteremia. At this threshold, the test characteristics were: sensitivity 64%, specificity 78%, positive predictive value 97%, and negative predictive value 15%. To enhance sensitivity, we reduced the ICU stay cutoff to ≥8 days. Under this revised classification, bacteremia occurring within the first 7 days of ICU admission was categorized as ESBL-producing Enterobacterales bacteremia, while cases developing on or after day 8 were classified as CRE bacteremia. This cutoff demonstrated 81% sensitivity and 56% specificity, with a strong positive predictive value of 96% but limited negative predictive value of 19%.

According to our hospital’s sepsis guidelines, when carbapenem resistance is suspected, empirical antibiotic options should include combination therapies containing either colistin/polymyxin B or amikacin. Among patients who developed CRE bacteremia in ICU, 35 (56.5%) had received these treatments. If ICU length of stay (8 or 13 d) had been used as a criterion for initiating CRE-active therapy, this number would have increased to 55 (88.7%) when using 8 days and to 51 (82.3%) when using 13 days as the threshold. This approach would have resulted unnecessary broad-spectrum coverage with polymyxins or amikacin in four (36.3%) and two (18.2%) patients in the control group who did not receive unnecessary colistin or amikacin treatment, respectively.

Recent evidence suggests carbapenem-resistant Gram-negative bacterial infections in low- and middle-income countries are often missed and inadequately managed due to limited diagnostic capacity with the weighted case fatality rate reaching 32% in bloodstream infections.^7^ This underscores the importance of appropriately targeting empiric therapy for at-risk populations. For ICU patients with unknown antibiotic exposure but comparable risk factors for antimicrobial resistance, the duration of ICU admission may serve as a practical predictor for CRE coverage needs. When other risk factors are known, ICU length of stay should be evaluated alongside them to guide empirical treatment for carbapenem-resistant infections. In the absence of other known risks, it may serve as the sole predictor. Our analysis supports using two distinct thresholds: while the 13-day cutoff provides greater specificity, the 8-day cutoff offers superior sensitivity (81%) and would be clinically preferable to avoid delays in initiating CRE-active therapy.

We have to acknowledge some limitations. The retrospective nature of this investigation limited our ability to collect reliable data on the use of invasive devices, a known risk factor. Additionally, the generalizability of our findings on CRE bacteremia prevalence and time to acquisition may be constrained by inter-center variability. Consequently, the proposed duration of stay threshold is intended not as a definitive mandate but as a pragmatic tool to aid empirical therapy decisions in critically ill patients. We suggest this approach is most valuable for tertiary referral centers with a high volume of critically ill transfers. Future studies are needed to validate these findings, establish context of specific cutoffs or to develop models allowing individual centers to calculate their own optimal duration based on local risk factors.
